# Tobacco smoking and all-cause mortality in a large Australian cohort study: findings from a mature epidemic with current low smoking prevalence

**DOI:** 10.1186/s12916-015-0281-z

**Published:** 2015-02-24

**Authors:** Emily Banks, Grace Joshy, Marianne F Weber, Bette Liu, Robert Grenfell, Sam Egger, Ellie Paige, Alan D Lopez, Freddy Sitas, Valerie Beral

**Affiliations:** National Centre for Epidemiology and Population Health, Australian National University, 62 Mills Road, Acton, ACT 2601 Australia; The Sax Institute, PO Box K617, Haymarket, Sydney, NSW 1240 Australia; Cancer Council NSW, PO Box 572, Kings Cross, Sydney, NSW 1340 Australia; School of Population Health, Edward Ford Building, University of Sydney, Sydney, NSW 2006 Australia; School of Public Health and Community Medicine, UNSW, Sydney, NSW 2052 Australia; National Heart Foundation of Australia, Level 12/500 Collins Street, Melbourne, VIC 3000 Australia; Melbourne School of Population and Global Health, University of Melbourne, Level 4, 207 Bouverie Street, Melbourne, VIC 3010 Australia; Cancer Epidemiology Unit, University of Oxford, Richard Doll Building, Roosevelt Drive, Oxford, OX3 7LF UK

**Keywords:** Cohort, Mortality, Smoking

## Abstract

**Background:**

The smoking epidemic in Australia is characterised by historic levels of prolonged smoking, heavy smoking, very high levels of long-term cessation, and low current smoking prevalence, with 13% of adults reporting that they smoked daily in 2013. Large-scale quantitative evidence on the relationship of tobacco smoking to mortality in Australia is not available despite the potential to provide independent international evidence about the contemporary risks of smoking.

**Methods:**

This is a prospective study of 204,953 individuals aged ≥45 years sampled from the general population of New South Wales, Australia, who joined the 45 and Up Study from 2006–2009, with linked questionnaire, hospitalisation, and mortality data to mid-2012 and with no history of cancer (other than melanoma and non-melanoma skin cancer), heart disease, stroke, or thrombosis. Hazard ratios (described here as relative risks, RRs) for all-cause mortality among current and past smokers compared to never-smokers were estimated, adjusting for age, education, income, region of residence, alcohol, and body mass index.

**Results:**

Overall, 5,593 deaths accrued during follow-up (874,120 person-years; mean: 4.26 years); 7.7% of participants were current smokers and 34.1% past smokers at baseline. Compared to never-smokers, the adjusted RR (95% CI) of mortality was 2.96 (2.69–3.25) in current smokers and was similar in men (2.82 (2.49–3.19)) and women (3.08 (2.63–3.60)) and according to birth cohort. Mortality RRs increased with increasing smoking intensity, with around two- and four-fold increases in mortality in current smokers of ≤14 (mean 10/day) and ≥25 cigarettes/day, respectively, compared to never-smokers. Among past smokers, mortality diminished gradually with increasing time since cessation and did not differ significantly from never-smokers in those quitting prior to age 45. Current smokers are estimated to die an average of 10 years earlier than non-smokers.

**Conclusions:**

In Australia, up to two-thirds of deaths in current smokers can be attributed to smoking. Cessation reduces mortality compared with continuing to smoke, with cessation earlier in life resulting in greater reductions.

**Electronic supplementary material:**

The online version of this article (doi:10.1186/s12916-015-0281-z) contains supplementary material, which is available to authorized users.

## Background

The risks of cancer, cardiovascular disease, respiratory disease, and a range of other health problems are increased in tobacco smokers and, as a consequence, smokers are more likely than non-smokers to die prematurely [1]. Smoking is a leading cause of morbidity and mortality in virtually every country in the world and is second only to high blood pressure as a risk factor for global disease burden [2]. It is arguably the leading readily preventable factor.

The relative risks of adverse health effects increase with increasing intensity of smoking, measured by the amount of tobacco smoked per day, and with increasing duration of smoking [3]. Smoking cessation imparts significant health benefits [3]. The overall effects of smoking on mortality in a population relate closely to the prevalence of current and past smoking and to the duration and intensity of smoking, among smokers. These indices relate, in turn, to the factors influencing smoking behaviour, including the stage of the smoking epidemic in the population under examination, to the relative success of tobacco control measures and to cultural and socioeconomic factors.

**Figure 1:**
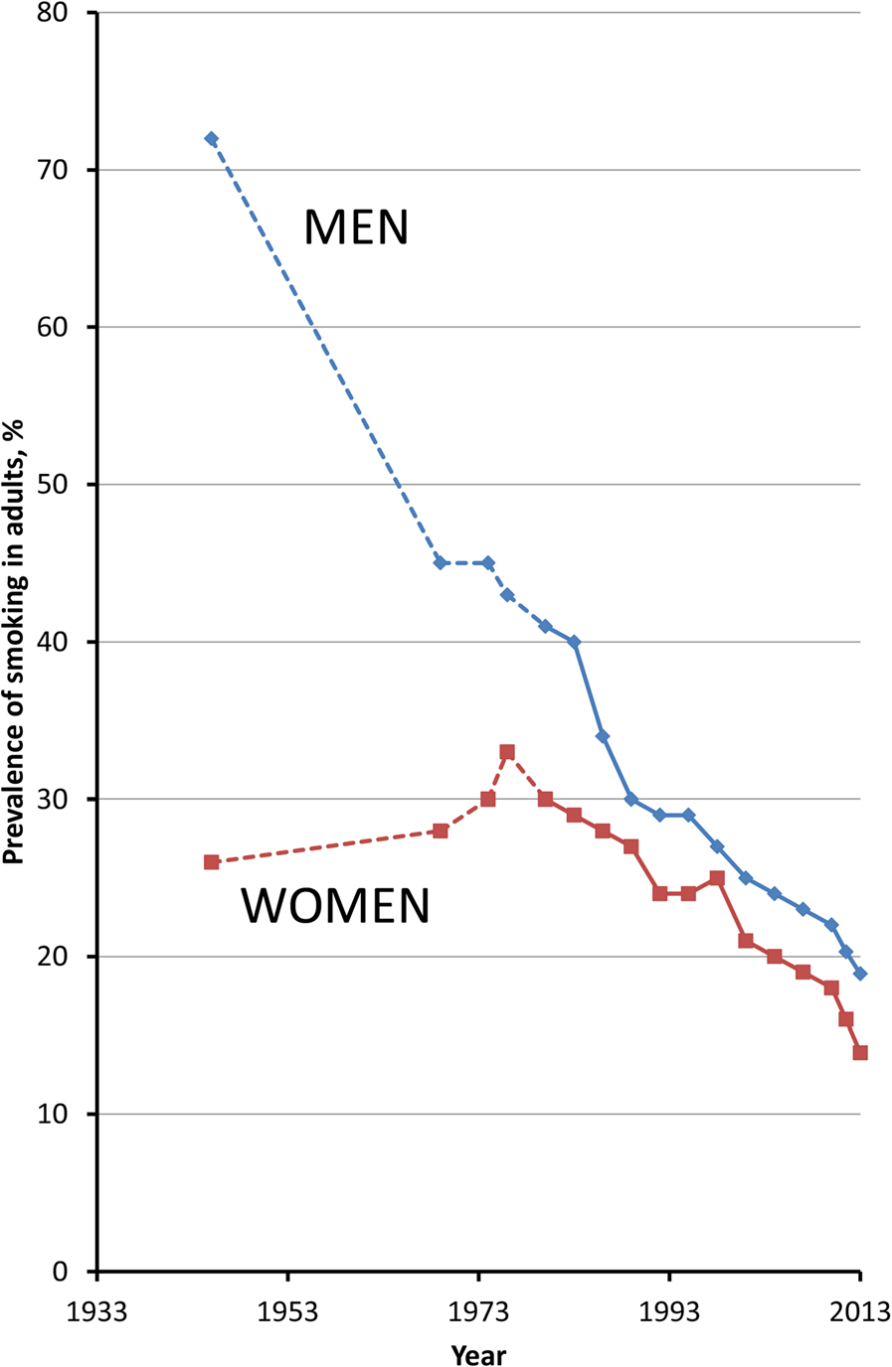
**Prevalence of current tobacco smoking among Australian adults, 1945–2013.** Data are from Scollo MM and Winstanley MH (1945–2010) [9], the Australian Health Survey (2011–2012) [10], and the National Drug Strategy Household Survey (2013) [8]. *Prior to 2001, the prevalences indicate those describing themselves as “current smokers”; from 2001–2010 the prevalences indicate those smoking daily or at least weekly. In 2011/2012 and 2013, they relate to current smoking, including daily, weekly, or less than weekly smoking. Data prior to 1980 are considered less reliable than from subsequent years and are represented with a dotted line [9].

Hence, both the relative risks of mortality and the overall population impacts of smoking are not uniform across the world and may also vary across time, population groups, and birth cohorts within a single location [3-5]. Large-scale quantitative evidence on the relationship of tobacco smoking to mortality among countries with a mature smoking epidemic is accruing, but is not yet available for Australia. In common with many countries, Australia has relied on the findings from studies conducted in the UK and US, including the British Doctors Study [3] and the American Cancer Society Cancer Prevention Studies [6], to underpin estimation of the population impact of smoking [7]. As well as providing local evidence, large-scale data from Australia have the potential to contribute to knowledge internationally by providing additional independent data on the effects of prolonged, heavy, and widespread smoking. Furthermore, Australia has been among the most successful nations regarding tobacco control, with most recent data indicating that 13% of adults in Australia reported daily smoking in 2013 [8]; data from Australia are therefore likely to provide insights into the risks of smoking in settings with high historical prevalence of smoking and low current prevalence (Figure 1).

This study aims to investigate the relationship of smoking to all-cause mortality in Australia, in the 45 and Up Study cohort. Although cause-specific mortality data have been used in analyses from other countries, these were not available for Australia at the time of writing. Participants in this population-based cohort study were predominantly born between 1920 and 1964, and have lived through the peak of the smoking epidemic, as well as through many changes in tobacco policy, legislation, and health information.

## Methods

The Sax Institute’s 45 and Up Study is an Australian cohort study of 267,153 men and women aged 45 and over, randomly sampled from the general population of New South Wales (NSW), Australia. Individuals joined the study by completing a postal questionnaire (distributed from 1 January 2006 to 31 December 2008) and giving informed consent for follow-up through repeated data collection and linkage of their data to population health databases. The study methods are described in detail elsewhere [11].

Baseline questionnaire data included information on socio-demographic factors, health behaviours, height and body weight, medical and surgical history, functional capacity, and physical activity. The study questionnaire is available online [12]. To provide data to allow correction for regression dilution, repeat data on smoking status were taken from a resurvey of a sample of 60,404 participants a mean of 3.3 years after recruitment.

Questionnaire data from study participants were linked probabilistically to data from the NSW Register of Births, Deaths and Marriages up to 30 June 2012 to provide data on fact and date of death. This probabilistic matching is known to be highly accurate (false-positive and false-negative rates <0.4%) [13]. Death registrations capture all deaths in NSW. Cause of death information was not available at the time of analysis. In order to conduct sensitivity analyses, questionnaire data were also linked probabilistically to data from the NSW Admitted Patient Data Collection, which is a complete census of all public and private hospital admissions in NSW. The linked data that were used contained details of admissions in participants from the year 2000 up to the point of recruitment, including the primary reason for admission using the International Classification of Diseases 10^th^ revision – Australian Modification (ICD-10-AM) [14] and up to 54 additional clinical diagnoses.

### Statistical methods

There were 266,777 participants with valid data on age and date of recruitment. Participants with data linkage errors (n = 20, 0.01%), age below 45 years at baseline (n = 3, 0.001%), and missing or invalid data on smoking status (n = 860, 3%) were excluded. To minimise the potential impact of changes in smoking behaviour and higher mortality in those with baseline illness (also known as reverse causality or the “sick quitter” effect), participants with a self-reported history of doctor-diagnosed cancer other than melanoma and/or non-melanoma skin cancer (n = 30,393, 11%) and those with a history of cardiovascular disease at baseline, defined as self-reported doctor-diagnosed heart disease, stroke, or blood clot on the baseline questionnaire (n = 30,548, 11%) were excluded from this study. It was not possible to exclude all individuals with respiratory illness because this information was not available in an appropriate form from the baseline questionnaire. However, sensitivity analyses were conducted to investigate the impact on the main results of additional exclusion of individuals with a history of admission to hospital with chronic obstructive pulmonary disease or other respiratory illnesses (defined as an admission to hospital with ICD-10-AM diagnosis codes J40 to J44 and J47 in any of the 55 diagnostic fields) in the 6 years prior to completing the baseline 45 and Up Study questionnaire.

Smoking status was classified according to the responses to the following series of items on the baseline questionnaire: “Have you ever been a regular smoker? If “Yes”, how old were you when you started smoking regularly? Are you a smoker now? If not, how old were you when you stopped smoking regularly? About how much do you/did you smoke on average each day?” Never-smokers were participants who answered “No” to the question, “Have you ever been a regular smoker?”; current smokers were those who answered “Yes” to this question and “Yes” to being a smoker now; and past smokers were those who indicated that they had ever been a regular smoker but who indicated that they were not a smoker now. The age at ceasing smoking, among past smokers, was taken as the age they indicated they stopped smoking regularly and was categorised as <25, 25–34, 35–44, 45–54, and ≥55 years. Among current and past smokers, the number of cigarettes smoked per day was taken from the answer to the question about how much they smoked on average each day and was categorised as ≤14, 15–24, and ≥25 cigarettes/day.

Mortality rates since baseline and 95% confidence intervals (CIs) were calculated for participants who reported being current, past, and never-smokers at baseline; these were indirectly standardised for age to the person-year distribution of the whole cohort population [15], and were presented separately for men and women. Hazard ratios (which are equivalent to, and described here as relative risks [RRs]) for mortality in men and women were estimated separately for men and women and according to birth cohorts with sufficient amounts of data, using Cox regression modelling, in which the underlying time variable was age. Estimates are shown initially accounting for age only (automatically adjusted for as the underlying time variable). Models are then presented adjusted for additional covariates derived from baseline questionnaire and participant location data, including education (<secondary school, secondary school graduation, trade/apprenticeship/certificate/diploma, university graduate); annual pre-tax household income (AUD <$20,000, $20,000–$39,999, $40,000–$69,999, ≥$70,000); region of residence (major cities, inner regional areas, outer regional/remote areas); alcohol consumption (0, 1–14, ≥15 alcoholic drinks/week), and body mass index (BMI) (<20, 20–24.99, 25–29.99, ≥30 kg/m^2^). Missing values for covariates other than smoking status were included in the models as separate categories. Hypertension and dyslipidaemia were considered likely to be part of the causal pathway between smoking and mortality and were not adjusted for. Sensitivity analyses were conducted: i) adjusting additionally for physical activity; and ii) categorising current smokers as those who reported being current smokers at baseline and past smokers who had ceased smoking 3 or fewer years prior to baseline.

Among current and never-smokers at recruitment, mortality rates and RRs by amount smoked were calculated according to categories of consumption reported at recruitment (≤14, 15–24, and ≥25 cigarettes/day). Mortality rates were then plotted against the mean number of cigarettes within each category reported at the 3-year resurvey among those who reported being current smokers at resurvey, as this was considered the best estimate of long-term mean consumption among all in that category, before the study started (Additional file 1: Table S1). Rates in never-smokers were plotted against the “0” on the x-axis. The RR of dying during the follow-up period was then quantified among past versus never-smokers, in those ceasing smoking at ages <25, 25–34, 35–44, and 45–54 years. Sensitivity analyses were conducted restricting the data to individuals aged ≥55 years, ensuring that all participants had the opportunity to quit at these ages.

The proportionality assumption of the Cox regression models was verified by plotting the Schoenfeld residuals against the time variable in each model, with a stratified form or time-dependent form of the model used where covariates displayed non-proportionality of hazards. No violations of the proportionality assumption were detected for the main exposure. Minor violations were observed in covariates for certain models and a stratified Cox model was fitted, as follows: overall analyses of current and past versus never-smokers – model stratified by education; analyses relating to birth decade – model stratified by alcohol, education, and income; analyses relating to number of cigarettes smoked per day – model stratified by income; analyses relating to age at smoking cessation – model stratified by alcohol and education.

Separately for males and females, absolute mortality rates for Australian smokers and non-smokers for age group *i* (45–54, 55–64, and 65–74 years) were estimated by M_i_/(1 + (RR − 1)P_*i*_) for non-smokers and RR times this for smokers [16] (where M_i_ and ${\mathrm{P}}_i$ represent 2010/2011 Australian population mortality rates and smoking prevalence estimated from other sources, respectively [17,18], and RR represents all-cause current smoker versus never-smoker RRs estimated in the current study). From these rates, cumulative risks of death for non-smokers and smokers at age *x* (55, 65, or 75 years) from age 45 were estimated by $1 - \exp \Big(-10{\displaystyle {\sum}_{\mathrm{i}=\left(45-54\right)}^{\mathrm{x}}{\mathrm{MR}}_{\mathrm{i}}\Big)}$ (where MR_i_ is either the smoker or non-smoker mortality rate for age group *i*) [19].

All statistical tests were two-sided, using a significance level of 5%. Analyses were carried out using SAS® version 9.3 [20] and Stata® versions 11 and 13.

Ethical approval for the 45 and Up Study as a whole was provided by the University of New South Wales Human Research Ethics Committee and specifically for this study by the NSW Population and Health Services Research Ethics Committee and the Australian National University Human Research Ethics Committee.

### Role of funding sources

The sponsors of this study had no role in study design, data collection, data analysis, data interpretation, or the writing of the report. All authors had full access to the data in the study and had final responsibility for the decision to submit for publication.

## Results

At baseline, 7.7% of the 204,953 study participants reported being current smokers and 34.1% were past smokers. Of the 84,312 participants with relevant data, 81,179 (96%) smoked only cigarettes, 1,572 (2%) smoked only pipes/cigars, and 1,561 (2%) reported smoking both. The prevalence of smoking was similar in men and women. Compared to never-smokers, current smokers were, on average, younger, less likely to be urban residents, of lower income and education level, and less likely to hold private health insurance; they were more likely to report consuming ≥15 alcoholic drinks/week and to have a BMI <20 kg/m^2^ (Table 1).

**Table 1:** **Characteristics of participants in the study according to smoking status**

	**Smoking status**			**Total**
	**Current**	**Past**	**Never**	
Total	15,768	69,900	119,285	204,953
Men	7,625 (48%)	37,335 (53%)	45,251 (38%)	90,211 (44%)
Age				
45–64 years	12,951 (82%)	45,107 (65%)	79,667 (67%)	137,725 (67%)
65–79 years	2,443 (15%)	19,378 (28%)	29,913 (25%)	51,734 (25%)
≥80 years	374 (2%)	5,415 (8%)	9,705 (8%)	15,494 (8%)
Residing in Major Cities	6,428 (41%)	30,103 (43%)	55,300 (46%)	91,831 (45%)
University degree	2,209 (14%)	15,300 (22%)	32,721 (27%)	50,230 (25%)
Household income ≥ $70,000	2,789 (18%)	18,218 (26%)	33,195 (28%)	54,202 (26%)
Private health insurance	6,714 (43%)	45,066 (64%)	84,007 (70%)	135,787 (66%)
≥15 alcoholic drinks/week	3,762 (24%)	15,685 (22%)	9,699 (8%)	29,146 (14%)
Highest physical activity tertile	5,278 (33%)	25,475 (36%)	39,044 (33%)	69,797 (34%)
Born in Australia	11,714 (74%)	50,845 (73%)	90,477 (76%)	153,036 (75%)
Body mass index				
<20 kg/m^2^	1,011 (6%)	1,848 (3%)	4,711 (4%)	7,570 (4%)
≥30 kg/m^2^	3,084 (20%)	16,160 (23%)	22,618 (19%)	41,862 (20%)

The mean age at commencing smoking was similar for male study participants born in the decades from 1920–1929 to 1960–1969 (Additional file 2: Table S2). For women, the average age at commencing smoking decreased from 24 years in those born in 1920–1929 to 17 years among those born in 1960–1969, similar to males born in this decade (Additional file 2: Table S2). The average duration of smoking in current smokers was 38.5 years (SD, 9.4 years), with the majority having smoked for 35 or more years and reporting consuming 15 or more cigarettes per day (Table 2). Because of the narrow age range of commencing smoking, duration of smoking among current smokers was strongly correlated with current age (r = 0.8). Data from the 3-year resurvey indicated consistency of reporting of never-smoker and ex-smoker status, with little misclassification and very few indicating that they had taken up smoking between surveys (Additional file 1: Table S1). Among current smokers at baseline who completed the 3-year resurvey, around one-third indicated that they were no longer smoking at resurvey, with those smoking fewer cigarettes per day being more likely to quit (Additional file 1: Table S1).

**Table 2:** **Smoking habits among current and former smokers, by sex**

		**Men**	**Women**
**Never-smoker**		45,251 (50%)	74,034 (65%)
**Current smoker**		7,625 (8%)	8,143 (7%)
Smoking duration (years)			
	Mean ± SD	39.9 ± 9.8	37.1 ± 8.8
	<20	124 (1.6%)	205 (2.5%)
	20–34	2,143 (28%)	3,085 (38%)
	35–49	3,869 (51%)	3,814 (47%)
	≥50	1,063 (14%)	541 (7%)
Cigarettes/day			
	Mean ± SD	18.9 ± 10.4	16.6 ± 8.6
	≤14	2,308 (30%)	3,016 (37%)
	15–24	2,931 (38%)	3,324 (41%)
	≥25	2,222 (29%)	1,632 (20%)
Age at starting smoking (years)			
	Mean ± SD	17.6 ± 5.2	18.7 ± 6.1
	<13	525 (7%)	222 (3%)
	13–17	3,741 (49%)	3,736 (46%)
	18–25	2,408 (32%)	2,863 (35%)
	≥25	526 (7%)	824 (10%)
**Past smoker**		37,335 (41%)	32,565 (28%)
Smoking duration (years)			
	Mean ± SD	22.6 ± 12.7	19.8 ± 12.2
	<20	15,669 (42%)	16,305 (50%)
	20–34	12,707 (34%)	10,126 (31%)
	35–49	5,767 (15%)	3,796 (12%)
	≥50	888 (2%)	351 (1%)
Cigarettes/day			
	Mean ± SD	20.1 ± 13.8	15.2 ± 10.4
	≤14	12,083 (32%)	16,121 (50%)
	15–24	15,004 (40%)	11,036 (34%)
	≥25	9,725 (26%)	4,910 (15%)
Age at starting smoking (years)			
	Mean ± SD	17.5 ± 3.8	18.5 ± 4.7
	<13	1,439 (4%)	469 (1%)
	13–17	18,316 (49%)	13,624 (42%)
	18–25	14,238 (38%)	14,363 (44%)
	≥25	1,621 (4%)	2,600 (8%)
Age at ceasing smoking (years)			
	Mean ± SD	40.1 ± 12.6	38.4 ± 12.5
	<25	3,421 (9%)	4,109 (13%)
	25–34	9,616 (26%)	9,207 (28%)
	35–44	9,548 (26%)	7,461 (23%)
	45–54	7,627 (20%)	6,354 (20%)
	≥55	5,259 (14%)	3,776 (12%)

Numbers may not add up to total due to missing data.

**Figure 2:**
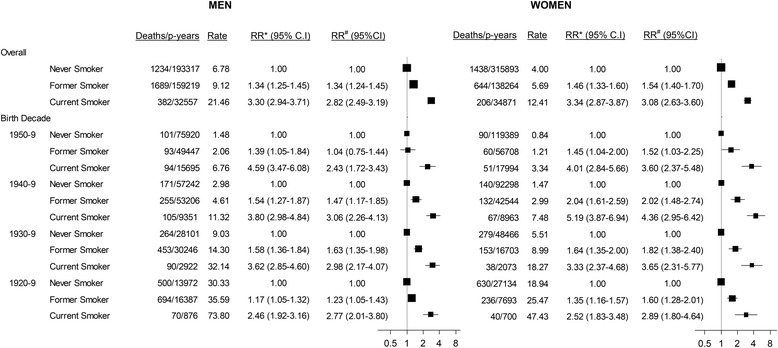
**Relative risks and absolute rates of all-cause mortality in the 45 and Up Study in current and past smokers relative to never-smokers, overall and by decade of birth.** Rate/1,000 person-years, indirectly standardised for age using the whole cohort distribution. *RR adjusted for age only (underlying time variable). ^#^RR adjusted for age, region of residence (major cities, inner regional areas, remote areas), alcohol consumption (0, 1–14, ≥15 drinks/week), annual pre-tax household income (AUD <$20,000, $20,000-$39,999, $40,000-$69,999, ≥$70,000), education (<secondary school, secondary school graduation, certificate or diploma, university graduate), and BMI (<20, 20–24.9, 25–29.9, ≥30). RRs are plotted on a log-scale and are represented with squares with areas inversely proportional to the variance of the logarithm of the RR, providing an indication of the amount of statistical information available; 95% CIs are indicated by horizontal lines.

Over a mean follow-up time of 4.26 years, 874,120 person-years accrued and 5,593 deaths occurred. The RR (95% CI) of dying during the follow-up period, adjusting for age, socioeconomic factors, alcohol intake, and BMI, was 2.96 (2.69–3.25) in current versus never-smokers overall, and 2.82 (2.49–3.19) and 3.08 (2.63–3.60) in men and women, respectively (Figure 2). The adjusted RRs in past versus never-smokers were 1.43 (1.35–1.52) overall and 1.34 (1.24–1.45) and 1.54 (1.40–1.70) in men and women, respectively (Figure 2). Although the absolute rates of death were higher for men than for women, the RRs relating to current and past smoking did not differ substantively between the sexes; nor did they vary materially according to birth cohort, from 1920–1959 (Figure 2). The results remained similar following exclusion of individuals with a history of admission to hospital with a diagnosis of chronic obstructive pulmonary disease and other respiratory illness; compared to never-smokers, RRs of mortality were 2.76 (2.42–3.14) and 2.95 (2.50–3.49) in male and female current smokers, respectively, with corresponding RRs in past smokers of 1.27 (1.17–1.37) and 1.39 (1.25–1.55). RRs did not change materially when further adjusted for physical activity and when data among past smokers were restricted to individuals aged 55 and over who had the opportunity to cease smoking from age 45–54 (data not shown). Nor did the RRs for mortality in current and former versus never-smokers change substantially when current smokers were defined as individuals reporting current smoking at baseline or within 3 years prior to baseline (for men RR (95% CI) using the new definitions of current/recent versus never-smokers: 2.65 (2.36–2.96) and former versus never-smokers: 1.34 (1.24–1.44); the corresponding figures for women were 3.26 (2.84–3.75) and 1.47 (1.33–1.62)).

**Figure 3:**
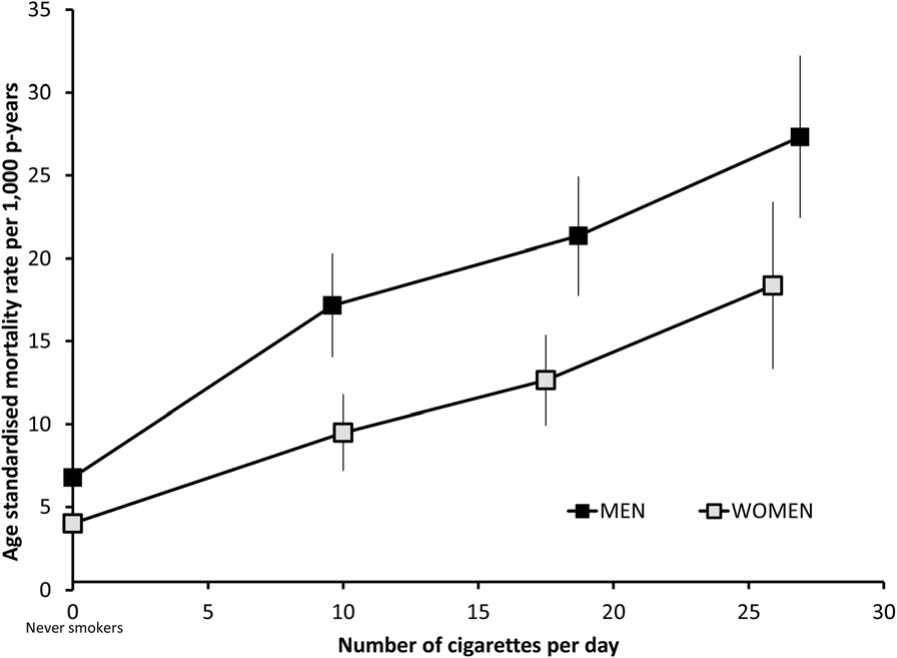
**Age standardised rates of all-cause mortality in current smokers and never-smokers, by smoking intensity.** Categories of smoking intensity (0 (never smokers), ≤14, 15–24, ≥25 cigarettes/day) are based on smoking behaviour reported at baseline. Rates are plotted against the mean number of cigarettes within each pre-defined category, based on smoking intensity reported at the 3-year resurvey among current smokers at resurvey, to minimise regression dilution bias. Vertical lines represent 95% confidence intervals; the intervals around the rates for never-smokers are small and contained within the squares that indicate the rates [Men 6.8 (6.4–7.2), Women 4.0 (3.8–4.2)].

Among current smokers, the mortality rate during the follow-up period increased markedly with increasing number of cigarettes smoked per day, with around a two-fold increase in mortality in the groups smoking 14 or fewer cigarettes per day (10 cigarettes per day, on average) and around a four-fold increase in the groups of current smokers who smoke ≥25 cigarettes per day, compared to never-smokers (Figure 3 and Additional file 3: Figure S1). While there was evidence that the increase in mortality with increasing numbers of cigarettes smoked was significantly greater for women than for men (*P*_(interaction)_ = 0.0002), the confidence intervals were relatively wide and the absolute mortality rates were considerably higher for men than for women.

**Figure 4:**
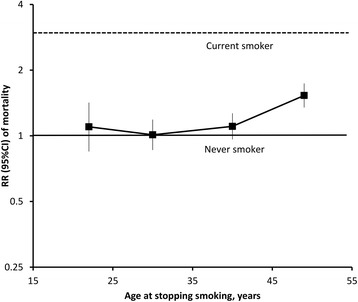
**Relative risk of all-cause mortality in past smokers relative to never-smokers in the 45 and Up Study, by age at smoking cessation.** RRs adjusted for age, sex, region of residence (major cities, inner regional areas, remote areas), alcohol consumption (0, 1–14, ≥15 drinks/week), annual pre-tax household income (AUD <$20,000, $20,000–$39,999, $40,000–$69,999, ≥$70,000), education (<secondary school, secondary school graduation, certificate or diploma, university graduate), and BMI (<20, 20–24.9, 25–29.9, ≥30). RRs plotted on log-scale against median value for age at stopping smoking categories <25, 25–34, 35–44, and 45–54 years.

The RR of dying during the follow-up period was 1.42 (1.29–1.58) among individuals ceasing smoking at age 45–54, compared to never-smokers (Figure 4); corresponding results were 1.36 (1.20–1.53) among men and 1.52 (1.27–1.82) among women (Additional file 4: Figure S2). Mortality diminished progressively with increasing time since cessation of smoking (data not shown) and did not differ significantly from that in never-smokers in individuals ceasing use prior to age 45 (Figure 4).

**Figure 5:**
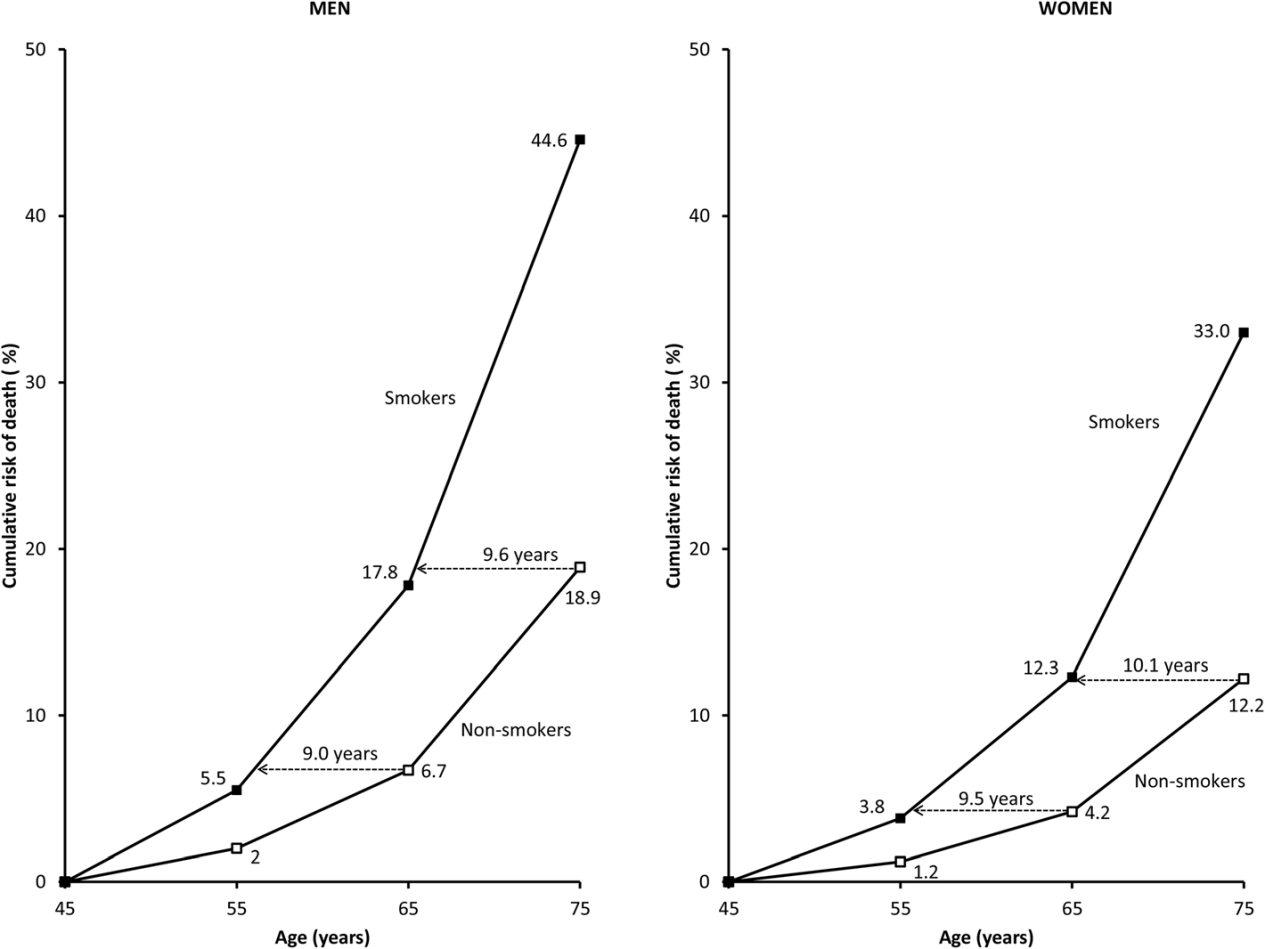
**Estimated cumulative risks of death from age 45 to 75 years in the Australian population in smokers and non-smokers for males and females.**

In Australia, male and female smokers were estimated to have the same risks of death 9.6 and 10.1 years earlier than 75-year-old non-smokers, respectively (Figure 5). Starting from age 45, 44.6% of male smokers in Australia would be estimated to die by age 75, compared to 18.9% of male non-smokers. Corresponding figures for females were 33.0% for smokers and 12.2% for non-smokers.

## Discussion

In this large-scale, population-based Australian study, death rates in current smokers were around three-fold those of people who had never smoked, in both men and women. On average, smokers died around 10 years earlier than non-smokers, over the ages examined. Mortality rates increased substantially with increasing intensity of smoking, with rates approximately doubling in those smoking around 10 cigarettes per day and four- to five-fold those of never-smokers in current smokers of 25 or more cigarettes per day. Cessation of smoking conferred large mortality benefits compared with continuing to smoke. These findings were adjusted for a range of potential confounding factors, including socioeconomic status, alcohol intake, and BMI.

These findings are virtually identical to those on the contemporary risks of smoking from the UK and US, where the RR of all-cause mortality in current versus never-smokers has been consistently reported at 2.8 to 3.0 [3,21-23] and similar to a recent report from Japan [24]. The finding of similar RRs among smokers across successive birth cohorts in this study indicates that, in common with these countries, it is likely that the full mortality impacts of smoking are being realised among smokers in Australia. The evolution of increasing smoking-attributable mortality over time is well documented, with RRs of all-cause mortality in current versus never-smokers of around 1.4 to 1.8 in the 1960s to 2.1 to 2.3 in the 1980s [3,6], corresponding to up to around one-third and one-half of the deaths in smokers being attributable to smoking, respectively. The findings from this and contemporary estimates from the US and UK indicate that up to two-thirds of deaths in smokers in the 21^st^ century in these settings are likely to have been caused by smoking [3,6,23]. The progressive increase in RRs has been attributed to the earlier commencement of smoking and greater intensity of smoking among successive birth cohorts, along with reductions in mortality among never-smokers [3,6,23]. In keeping with this, the smoking-related RRs in countries where widespread heavy and prolonged smoking from an early age began more recently are somewhat lower than those observed here [25].

The study provides the first large-scale direct evidence on the relationship of smoking to mortality in Australia. The population examined displays quantitatively many of the characteristics of a mature epidemic of smoking in the Western context, namely a relatively low prevalence of current smoking; similar prevalence of current smoking in men and women; long durations and stable intensities of smoking among current smokers; young and stable age at commencing smoking; a high prevalence of past smoking; and stable RRs of smoking-related mortality in successive birth cohorts [26]. Consistent RRs among successive birth cohorts were observed although the tar content in cigarettes in Australia has fallen over the last four decades [9]. The findings also demonstrate the continuing harms of smoking, despite highly successful tobacco control measures, and the need for continuing attention and control. The introduction of “plain packaging” for cigarettes in Australia in 2012 is an example of the continuing efforts required.

This study has the strength of being large and population-based, with independent and virtually complete data on the outcome of all-cause mortality. The study ascertained smoking status from questionnaire items that are based on those used in the Million Women Study, allowing direct international comparison of results [23]. Repeat data collection on smoking status allowed correction for regression dilution, such that the findings relating to smoking intensity are likely to reflect long-term habits. In keeping with the continuing decline in smoking prevalence in Australia, the data indicate that a substantial minority of current smokers at baseline ceased smoking during the follow-up period. This suggests that the estimated hazard ratios for mortality among current smokers at baseline are likely to be conservative. Although we do not have direct data on use of smokeless tobacco products among participants, importation and supply of these products has been illegal in Australia since 1991 and use has been negligible since then [27].

The study provides evidence on the effects of heavy and prolonged smoking in a setting where the prevalence of smoking is now low. Around 12% of individuals aged 45 and over in NSW were estimated to be current smokers at the time when the 45 and Up Study commenced [28] and, following exclusions, current smokers made up around 8% of the cohort. It should be noted that although the 45 and Up Study is, like the vast majority of cohort studies, not strictly representative of the general population, the results presented here are based on internal comparisons within the cohort and are likely to be reliable [28]. Moreover, as the British Doctors Study illustrates, cohort studies do not need to be representative to produce effect estimates that are generalizable. Follow-up time was relatively short, which has the advantage of meaning that smoking status measured at baseline is likely to broadly represent smoking status during the follow-up period. NSW is the most populous state in Australia, comprising around one-third of the total population. Smoking prevalence and cause-specific death rates for major causes of death in NSW are similar to those observed nationally [17,18].

To ensure that the study focussed on the likely causal effect of smoking on mortality, participants who had had cancer or cardiovascular disease at baseline were excluded. Although it was not possible to exclude individuals with chronic respiratory disease, sensitivity analyses indicated that the results did not change materially when individuals with a previous hospital admission including a diagnosis of respiratory illness were excluded. Because of the tendency for smokers, particularly older smokers, to quit due to ill-health, it was not possible to reliably estimate the mortality risks in those ceasing smoking at older ages (i.e., 55 years or older), although they represented the minority of past smokers. It should be noted that the findings here are contingent on surviving to age 45; however, few deaths attributable to smoking are likely to have occurred below this age.

The evidence presented here relates to death from any cause. Data on cause of death were not available at the time this study was conducted. International evidence shows that the vast majority of excess deaths in smokers are caused by smoking and are due to conditions such as cardiovascular disease, cancer, and chronic lung disease. However, it should be borne in mind that a minority of deaths, such as those related to suicide, may be increased in smokers but may not be wholly caused by smoking. Hence, although we are not able to exclude the relatively small number of deaths that are less likely to be causally related to smoking, the large majority of the observed excess mortality in smokers observed here would have been caused by smoking [3].

## Conclusions

The national prevalence of smoking in Australia has fallen rapidly and is now among the lowest in the world, with an estimated 13% of adults smoking daily. A number of countries are moving towards tobacco “eradication”. These data indicate that, in a low prevalence setting, the risks of continuing to smoke and the benefits of cessation remain high.

## Additional files

Additional file 1: Table S1,
**Smoking patterns at re-survey, by smoking status reported at baseline.**
Additional file 2: Table S2,
**Smoking habits among current and former smokers by sex and birth decade.**
Additional file 3: Figure S1,
**Relative risk (RR) of all-cause mortality in current and past smokers relative to never smokers, by smoking intensity, separately for men and women.** Rate/1,000 person-years, indirectly standardised for age using the whole cohort distribution. *RR adjusted for age only (underlying time variable). ^#^RR adjusted for age (underlying time variable), region of residence (major cities, inner regional areas, remote areas), alcohol consumption (0 , 1–14, ≥15 drinks/week), annual pre-tax household income (AUD <$20,000, $20,000–$39,999, $40,000–$69,999, ≥$70,000), education (<secondary school, secondary school graduation, certificate or diploma, university graduate), and BMI (<20, 20–25, 25–30, ≥30). RRs are plotted on a log-scale and are represented with squares with areas inversely proportional to the variance of the logarithm of the RR, providing an indication of the amount of statistical information available; 95% CIs are indicated by horizontal lines. Additional file 4: Figure S2,
**Relative risk (RR) of all-cause mortality in past smokers relative to never-smokers in the 45 and Up Study, by age at smoking cessation, separately for men and women.** Rate/1,000 person-years, indirectly standardised for age using the whole cohort distribution. *RR adjusted for age only (underlying time variable). ^#^RR adjusted for age (underlying time variable), region of residence (major cities, inner regional areas, remote areas), alcohol consumption (0, 1–14, ≥15 drinks/week), annual pre-tax household income (AUD <$20,000, $20,000–$39,999, $40,000–$69,999, ≥$70,000), education (<secondary school, secondary school graduation, certificate or diploma, university graduate), and BMI (<20, 20–25, 25–30, ≥30). RRs are plotted on a log-scale and are represented with squares with areas inversely proportional to the variance of the logarithm of the RR, providing an indication of the amount of statistical information available; 95% CIs are indicated by horizontal lines. Mean (±SD) number of cigarettes/day for the above age at smoking cessation groups (<25, 25–35, 35–44, 45–54 years) were: 15.4 ± 10.5, 18.7 ± 12.5, 21 ± 14.5, and 21.8 ± 14.6, respectively, among men, and 11.7 ± 8.4, 13.8 ± 9.2, 16.1 ± 10.8, and 17.2 ± 11.3, respectively, among women.

## Abbreviations

AUD: Australian dollars
BMI: Body mass index
CIs: Confidence intervals
ICD-10-AM: International Classification of Diseases 10^th^ revision – Australian Modification
NSW: New South Wales
RR: Hazard ratios (described here as relative risks)
