# Supplementary material for: Tobacco smoking and all-cause mortality in a large Australian cohort study: findings from a mature epidemic with current low smoking prevalence
Source: BMC Med. 2015 Feb 24;13:38. doi: 10.1186/s12916-015-0281-z (PMC4339244; doi:10.1186/s12916-015-0281-z)
Supplement: Additional file 1: Table S1, — Smoking patterns at re-survey, by smoking status reported at baseline. [file 12916_2015_281_MOESM1_ESM.pdf]

Supplementary Table 1: Smoking patterns at re-survey, by smoking status reported at baseline

	Never smoker at recruitment	Ex-smoker at recruitment	Current smoker at recruitment, by cigarettes per day			
**MEN**			≤14	15-24	≥25	All amounts*
Current smoker at re-survey (%)	<0·5% (47/13,790)	1·9% (237/12,483)	59.1% (296/501)	67·0% (377/563)	73·0% (359/492)	66.05 % (1,043/1,579)
Cigarettes smoked per day at resurvey (SD)	5·8(9·6)	21·2(14·8)	11·0 (5·6)	19·6 (7·4)	29·7 (11·2)	20·4 (11·3)
**WOMEN**						
Current smoker at re-survey (%)	<0·5% (66/21,010)	2·2% (205/9,240)	65·3% (445/682)	73·2% (525/717)	75·6% (298/394)	70·6% (1,284/1,820)
Cigarettes smoked per day at resurvey (SD)	3·4(5·4)	15·4(10·6)	10·1 (5·0)	17·7 (6·3)	27·1 (8·9)	16·9 (9·2)

SD standard deviation
*Numbers do not sum to total due to missing values
