# Supplementary material for: Tobacco smoking and all-cause mortality in a large Australian cohort study: findings from a mature epidemic with current low smoking prevalence
Source: BMC Med. 2015 Feb 24;13:38. doi: 10.1186/s12916-015-0281-z (PMC4339244; doi:10.1186/s12916-015-0281-z)
Supplement: Additional file 2: Table S2, — Smoking habits among current and former smokers by sex and birth decade. [file 12916_2015_281_MOESM2_ESM.pdf]

Supplementary Table 2: Smoking habits among current and former smokers by sex and birth decade

	Men					Women				
Birth decade	1920-9	1930-9	1940-9	1950-9	1960-9	1920-9	1930-9	1940-9	1950-9	1960-9
Current Smokers										
n	227	691	2161	3613	915	182	487	2057	4140	1261
Age at starting smoking (years)	18 ± 5	18 ± 6	18 ± 6	17 ± 5	17 ± 5	24 ± 12	23 ± 9	20 ± 6	18 ± 5	17 ± 5
Cigarettes/day	14 ± 8	19 ± 12	20 ± 10	19 ± 10	18 ± 9	14 ± 10	16 ± 8	17 ± 9	17 ± 9	16 ± 8
Smoking duration (years)	63 ± 5	54 ± 6	45 ± 6	36 ± 6	30 ± 5	58 ± 12	50 ± 9	43 ± 6	34 ± 6	30 ± 5
Former Smokers										
n	3928	7049	12356	11469	2167	1878	3882	9893	13242	3502
Age at starting smoking (years)	18 ± 4	18 ± 4	17 ± 4	17 ± 4	17 ± 4	22 ± 7	21 ± 6	19 ± 5	18 ± 4	17 ± 4
Cigarettes/day	18 ± 14	21 ± 15	21 ± 14	19 ± 12	18 ± 12	12 ± 9	14 ± 11	16 ± 11	15 ± 10	15 ± 10
Smoking duration (years)	29 ± 15	27 ± 14	23 ± 12	18 ± 10	16 ± 9	29 ± 15	26 ± 13	22 ± 12	17 ± 10	15 ± 9
Age at ceasing smoking (years)	48 ± 14	45 ± 13	40 ± 12	36 ± 10	34 ± 8	50 ± 15	47 ± 13	41 ± 12	34 ± 10	32 ± 8

Data are mean ± sd**.** There were 34 current smokers (18 men and 16 women) and 534 former smoker (366 men and 168 women) born before 1920, who are not included in this table.
