# Supplementary figures and images for: Tobacco smoking and all-cause mortality in a large Australian cohort study: findings from a mature epidemic with current low smoking prevalence
Source: BMC Med. 2015 Feb 24;13:38. doi: 10.1186/s12916-015-0281-z (PMC4339244; doi:10.1186/s12916-015-0281-z)

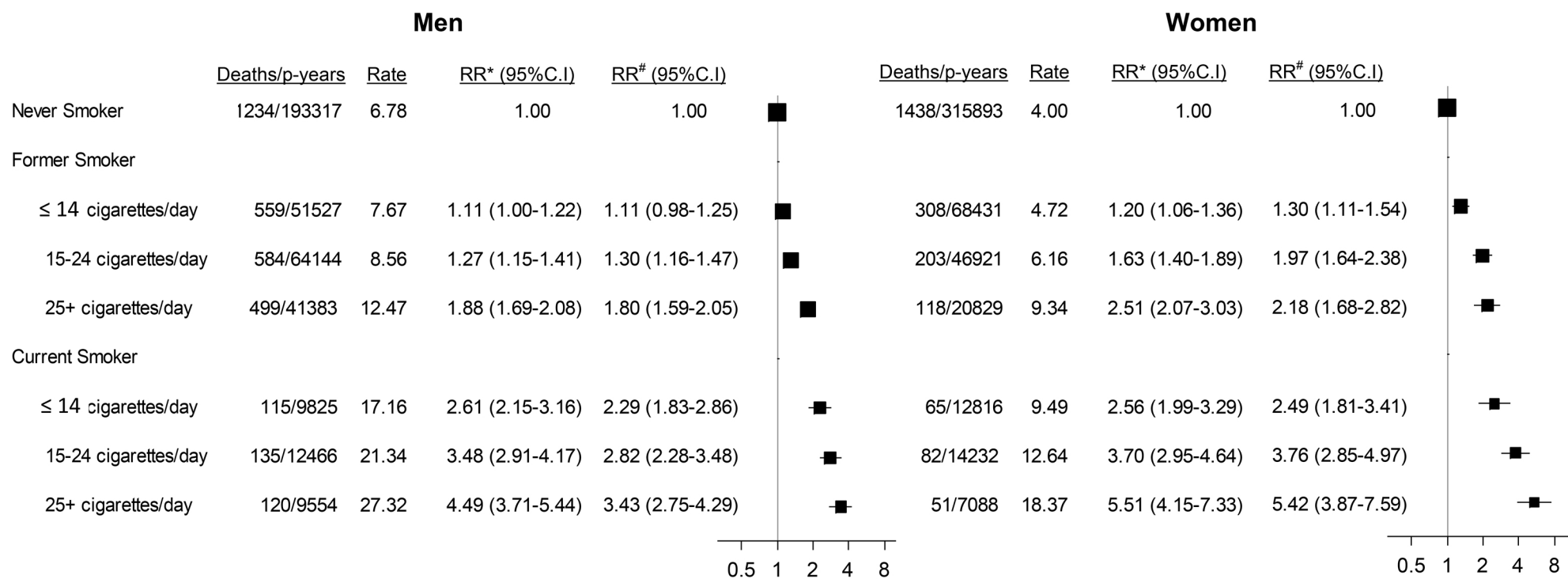

Supplement: Additional file 3: Figure S1, — Relative risk (RR) of all-cause mortality in current and past smokers relative to never smokers, by smoking intensity, separately for men and women. Rate/1,000 person-years, indirectly standardised for age using the whole cohort distribution. *RR adjusted for age only (underlying time variable). #RR adjusted for age (underlying time variable), region of residence (major cities, inner regional areas, remote areas), alcohol consumption (0 , 1–14, ≥15 drinks/week), annual pre-tax household income (AUD <$20,000, $20,000–$39,999, $40,000–$69,999, ≥$70,000), education (<secondary school, secondary school graduation, certificate or diploma, university graduate), and BMI (<20, 20–25, 25–30, ≥30). RRs are plotted on a log-scale and are represented with squares with areas inversely proportional to the variance of the logarithm of the RR, providing an indication of the amount of statistical information available; 95% CIs are indicated by horizontal lines. [file 12916_2015_281_MOESM3_ESM.pdf]

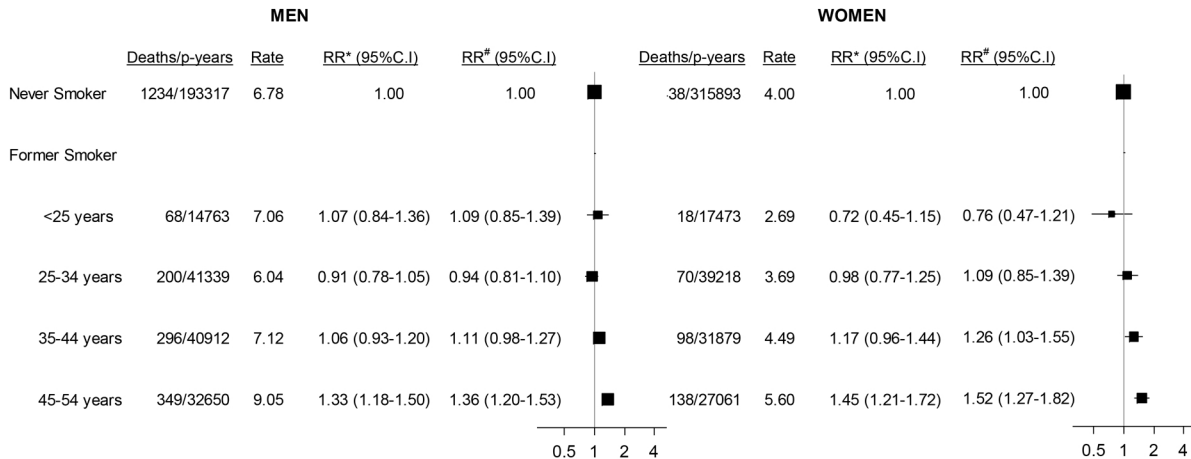

Supplement: Additional file 4: Figure S2, — Relative risk (RR) of all-cause mortality in past smokers relative to never-smokers in the 45 and Up Study, by age at smoking cessation, separately for men and women. Rate/1,000 person-years, indirectly standardised for age using the whole cohort distribution. *RR adjusted for age only (underlying time variable). #RR adjusted for age (underlying time variable), region of residence (major cities, inner regional areas, remote areas), alcohol consumption (0, 1–14, ≥15 drinks/week), annual pre-tax household income (AUD <$20,000, $20,000–$39,999, $40,000–$69,999, ≥$70,000), education (<secondary school, secondary school graduation, certificate or diploma, university graduate), and BMI (<20, 20–25, 25–30, ≥30). RRs are plotted on a log-scale and are represented with squares with areas inversely proportional to the variance of the logarithm of the RR, providing an indication of the amount of statistical information available; 95% CIs are indicated by horizontal lines. Mean (±SD) number of cigarettes/day for the above age at smoking cessation groups (<25, 25–35, 35–44, 45–54 years) were: 15.4 ± 10.5, 18.7 ± 12.5, 21 ± 14.5, and 21.8 ± 14.6, respectively, among men, and 11.7 ± 8.4, 13.8 ± 9.2, 16.1 ± 10.8, and 17.2 ± 11.3, respectively, among women. [file 12916_2015_281_MOESM4_ESM.pdf]
